# Supplementary material for: Highest fusion performance without harmful edge energy bursts in tokamak
Source: Nat Commun. 2024 May 11;15:3990. doi: 10.1038/s41467-024-48415-w (PMC11088687; doi:10.1038/s41467-024-48415-w)
Supplement: Supplementary file 1 — Supplementary information [file 41467_2024_48415_MOESM1_ESM.pdf]

Supplementary Information

Highest Fusion Performance without Harmful Edge Energy Bursts in Tokamak

S.K.Kim[1], R.Shousha[1], S.M.Yang[1], Q.Hu[1], S.H.Hahn[2], A.Jalalvand[3], J.-K.Park[4], N.C.Logan[5], A.O.Nelson[5], Y.-S.Na[4], R.Nazikian[6], R.Wilcox[7], R.Hong[8], T.Rhodes[8], C.Paz-Soldan[5], Y.M.Jeon[2], M.W.Kim[2], W.H.Ko[2], J.H.Lee[2], A.Battey[5], G.Yu[9], A.Bortolon[1], J.Snipes[1], and E.Kolemen[1,3,a)]

[1]Princeton Plasma Physics Laboratory, Princeton, U.S.A
[2]Korea Institute of Fusion Energy, Daejeon, South Korea
[3]Princeton University, Princeton, U.S.A
[4]Seoul National University, Seoul, South Korea
[5]Columbia University, New York, U.S.A
[6]General Atomics, San Diego, U.S.A
[7]Oak Ridge National Laboratory, Oak Ridge, U.S.A
[8]University of California Los Angeles, Los Angeles, U.S.A
[9]University of California Davis, Davis, U.S.A

This PDF file includes:
Supplementary Figures 1 and 2.

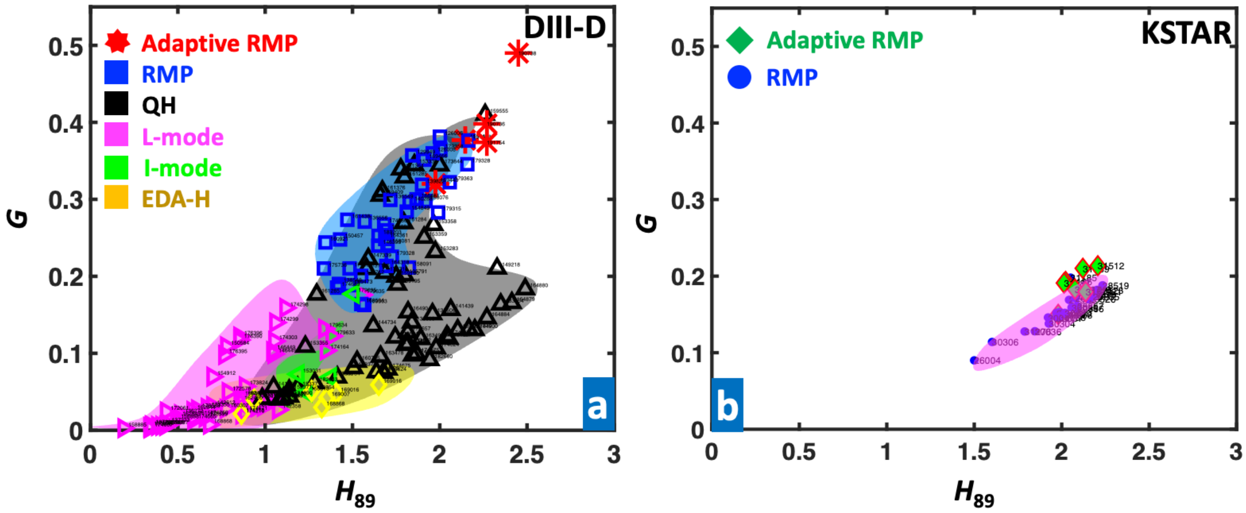

Supplementary Figure 1. **Performance comparison of ELM-free discharges in DIII-D and KSTAR.** Normalized energy confinement time (H_{89}) versus the figure of merit (G) at ELM-free state. These cover various RMP and non-ELMing scenarios, including QH, L-mode, I-mode, and EDA-H mode in DIII-D. The red star and green diamond markers show the adaptive RMP discharges in **a** DIII-D and **b** KSTAR, respectively. These databases cover the discharges through 2022.

a) Corresponding author: ekolemen@princeton.edu

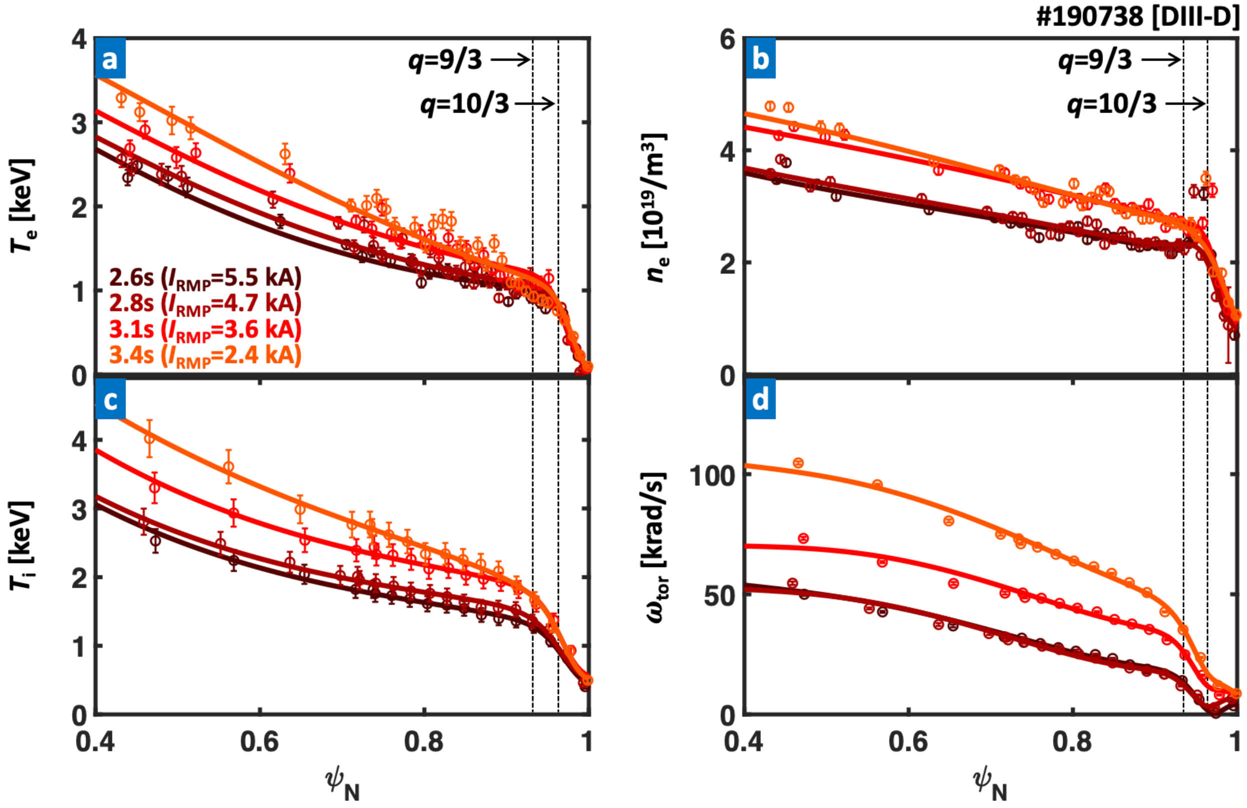

Supplementary Figure 2. **Plasma profiles of #190738 for RMP ramp-down phase at 2.6s (I_{RMP}=5.5 kA), 2.8s (I_{RMP} =4.7 kA), 3.1s (I_{RMP} =3.6 kA), and 3.4s (I_{RMP} =2.4 kA). a** Electron temperature (T_e), **b** density (n_e), **c** ion temperature (T_i), and **d** rotation (ω_{tor}) with statistical error bars. The error bars represent the 1-sigma uncertainty of the fit to the raw data. Here, ψ_N is normalized poloidal flux, and I_{RMP} is the RMP coil current. Ion temperature and rotation are measured by a charge-exchange recombination system for carbon (6+) impurities, assuming similar values with the main ion. Electron temperature and density are measured by the Thomson Scattering system. Vertical dotted lines show the location of q =9/3 and 10/3 surfaces, where q is a safety factor.

[a] Corresponding author: ekolemen@princeton.edu
